# Cardiovascular Pulsatility Increases in Visual Cortex Before Blood Oxygen Level Dependent Response During Stimulus

**DOI:** 10.3389/fnins.2022.836378

**Published:** 2022-02-03

**Authors:** Niko Huotari, Johanna Tuunanen, Lauri Raitamaa, Ville Raatikainen, Janne Kananen, Heta Helakari, Timo Tuovinen, Matti Järvelä, Vesa Kiviniemi, Vesa Korhonen

**Affiliations:** ^1^Oulu Functional Neuro Imaging Group, Research Unit of Medical Imaging Physics and Technology (MIPT), University of Oulu, Oulu, Finland; ^2^Department of Diagnostic Radiology, Medical Research Center (MRC), Oulu University Hospital, Oulu, Finland

**Keywords:** cardiovascular pulsations, magnetic resonance encephalography, fast fMRI, visual stimulation, task activation

## Abstract

The physiological pulsations that drive tissue fluid homeostasis are not well characterized during brain activation. Therefore, we used fast magnetic resonance encephalography (MREG) fMRI to measure full band (0–5 Hz) blood oxygen level-dependent (BOLD_FB_) signals during a dynamic visual task in 23 subjects. This revealed brain activity in the very low frequency (BOLD_VLF_) as well as in cardiac and respiratory bands. The cardiovascular hemodynamic envelope (CHe) signal correlated significantly with the visual BOLD_VLF_ response, considered as an independent signal source in the V1-V2 visual cortices. The CHe preceded the canonical BOLD_VLF_ response by an average of 1.3 (± 2.2) s. Physiologically, the observed CHe signal could mark increased regional cardiovascular pulsatility following vasodilation.

## Introduction

The earliest report of discernible physical reactions in human brain were based on observations by Angelo Mosso in patients with open skull lesions, who showed increased brain pulsatility during performance of cued visual and cognitive tasks (Mosso, 1880). Hyperemic reactions and marked pulsatile responses to direct electric brain stimulus were a decade later reported in animal models by Roy and Sherrington (1890). In addition, the EEG pioneer Hans Berger described from direct interoperative observations of brain surgery patients three predominant brain pulsations during neurosurgical procedures, i.e., the vasomotor (<0.1 Hz), respiratory (∼0.2 Hz), and cardiovascular (∼1 Hz) (Berger, 1901). Vasomotor waves are slow fluctuations of arterial smooth muscle tone that induce blood flow undulations, while cardiovascular pulsations are arterial pressure impulses induced by repeated heart beats.

In 1990, the advent of T2* weighted functional magnetic resonance imaging (fMRI) enabled the detection of hyperemic increases in cerebral blood flow coupled to regional neuronal activation as an increase in the signal intensity level (Ogawa and Lee, 1990; Bandettini et al., 1992; Kwong et al., 1992; Ogawa et al., 1992; Buxton, 2012). According to current understanding, activated neurons stimulate the regional neurovascular unit, triggering dilatation of regional pre-capillary sphincters, which increases pulsatile blood flow after a response delay of 3–6 s (Biswal et al., 2003; Kucewicz et al., 2007). The enhanced inflow of oxygenated blood balloons the cortical veins that drain the activated area, thus reducing the local paramagnetic deoxyhemoglobin concentration, in conjunction with an increased blood volume (Ogawa and Lee, 1990; Buxton, 2012). Together these changes in blood circulation lower T2* dephasing of regional (peri)vascular water proton spins, which is detectable as an increase in the blood oxygen level dependent (BOLD) signal intensity level downstream from activated areas (Bandettini et al., 1992; Ogawa et al., 1992; Buxton, 2012).

Importantly, the T2* weighted signal also contains information about other physiological phenomena, including the three physiological pulsations (Windischberger et al., 2002; Wise et al., 2004; Birn et al., 2006; Chang et al., 2008). Studies with fast fMRI sequences have recently succeeded in separating three primary physiological pulsation sources in human brain, namely very low frequency (VLF; < 0.1 Hz), respiratory related pulsations (0.1–0.5 Hz), and cardiovascular pulsations (0.8–1.5 Hz), along with their harmonic interactions (Posse et al., 2013; Rajna et al., 2015; Kiviniemi et al., 2016; Dreha-Kulaczewski et al., 2017; Huotari et al., 2019; Raitamaa et al., 2021).

As discovered by Nedergaard in 2013, these physiological pulsations play a key role in maintaining brain tissue homeostasis by driving the cerebrospinal fluid (CSF) mediated convection of solutes through the brain parenchyma (Iliff et al., 2012; Nedergaard, 2013; Kiviniemi et al., 2016; Ringstad and Eide, 2020). Mestre et al. (2018) have shown that cardiovascular brain pulsations are a key factor in convective clearance of waste molecules and metabolites along perivascular CSF conduits around the brain cortex. Furthermore, an earlier study shows that slow CSF pulsations are increased in sleep (Fultz et al., 2019). In rodent studies, the pulsatility of the blood vessel wall was directly proportional to the efficiency of CSF solute transport in perivascular spaces (Mestre et al., 2018). van Veluw et al. (2020) showed that a 5 s on/off visual stimulus induced a harmonic 0.1 Hz vasomotion, which seemingly increased clearance of a CSF tracer molecule from the brain interstitium. Furthermore, a recent paper linked the global BOLD signal and carotid pulsatility to the hemodynamic delays of the brain (Amemiya et al., 2020).

However, the effects of the physiological brain pulsations have not been comprehensively analyzed with respect to the temporal BOLD response dynamics that occur during brain activation. In this study, we tested the hypothesis that regional brain hyperemic activation responses should coincide with increased amplitude of physiological pulsations that are implicated as drivers of CSF circulation. To this end, we presented widefield visual stimulation to healthy volunteers and separately measured the amplitude of associated cardiovascular and respiratory brain pulsations in primary visual cortex (V1), aiming to establish a link between neuronal activation and increases in specific pulsation bands known previously to promote (para)vascular flow

## Materials and Methods

### Participants

25 healthy subjects (age: 27.6 ± 5.7 years, 13 females) were positioned in the MRI scanner and asked to lie still and keep their eyes open with gaze fixated on a screen. Participants wore ear plugs and cushions beside their ears to reduce their perception of scanner noise and to restrict head movement. The total scan duration was 4 min and 5 s. Data from two subjects were excluded from the analyses due to a combination of slightly head elevated movement and a limited BOLD response to the last (fourth) stimulus period, leaving 23 subjects (age: 27.2 ± 4.6 years, 12 females) for the analyses. Written informed consent was obtained from each subject prior to scanning, in accordance with the Helsinki declaration. The study protocol was approved by the Regional Ethics Committee of Northern Ostrobothnia Hospital District in Oulu University Hospital.

### Data Acquisition and Preprocessing

Subjects were imaged in a Siemens 3T SKYRA scanner using a 32-channel head-coil. A previously documented MRI-compatible multimodal neuroimaging system served to collect the datasets (Korhonen et al., 2014). We used a fast fMRI technique, namely magnetic resonance encephalography (MREG), as the scanning method. MREG is a 3D single shot “stack of spirals” sequence that under-samples k-space to allow critical imaging of physiological pulsations at a sampling rate of 10 Hz (Assländer et al., 2013). The following MREG scanning parameters were used: repetition time (TR = 100 ms), echo time (TE = 36 ms), field of view (FOV = 192 mm), 3D matrix = 64 mm^3^ (3 mm isotropic) and flip angle (FA = 5°).

The reference scan was used to estimate the head coil sensitivity profiles and to provide static off-resonance map used for off-resonance corrected reconstruction. Then the respective reference and raw MREG scan data was reconstructed using a MATLAB recon tool provided by the sequence developers on a multi-core Linux-computing cluster. Within the tool, L2-Tikhonov regularization was set to lambda = 0.15 rather than the default value of 0.2 to attain higher signal-to-noise ratio (SNR). Conjugate gradient optimization was performed using 35 iterations for increased robustness in the convergence of the images. Dynamic off-resonance correction in k-space was used to reduce motion-induced artifacts in the datasets (Pfeuffer et al., 2002; Zahneisen et al., 2014). Anatomical 3D MPRAGE (TR = 1,900 ms, TE = 2.49 ms, TI = 900 ms, FOV = 240 mm, 0.9 mm cubic voxel, flip angle = 9°) images were acquired to be used in MREG data anatomic registration.

MREG datasets passed through a typical FSL preprocessing pipeline (Jenkinson et al., 2012). The datasets were high pass filtered at a cutoff of 0.008 Hz (125 s). T1-relaxation effects were minimized using 8 s of dummy scans at the start of each scan. FSL MCFLIRT was used for motion correction. Datasets were spatially smoothed with a 5 mm full width and half maximum (FWHM) Gaussian kernel using *fslmaths*. FSL BET was used for brain extraction (fractional intensity = 0.25, threshold gradient = 0.22 with neck and bias-field correction). AFNI *3dDespike* was performed to remove spikes from the datasets. The MREG data were registered to 4 mm resolution anatomical 3D MPRAGE standard images in MNI space prior to group-level independent component analysis (ICA) *via* FSL MELODIC, which produced 70 independent components (ICs) (Jenkinson and Smith, 2001; Jenkinson et al., 2002).

### Visual Stimulation

**FIGURE 1:**
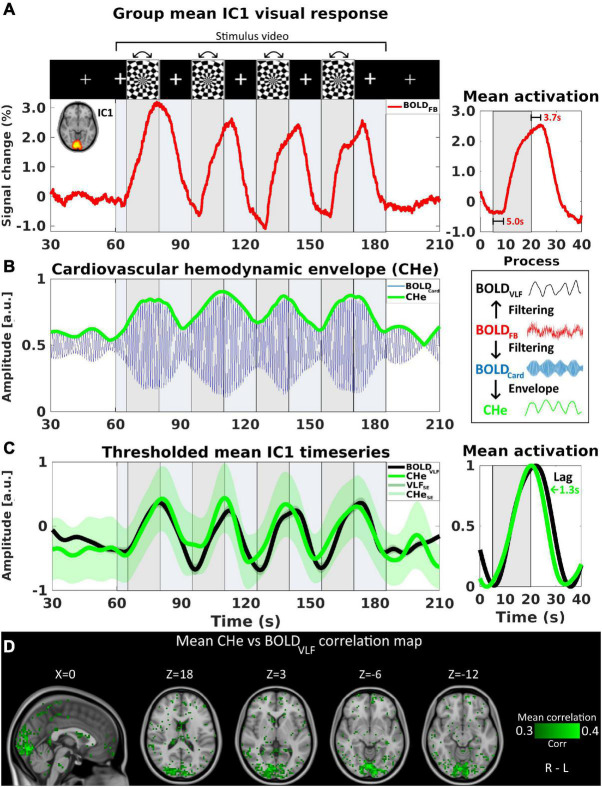
The cardiovascular hemodynamic envelope (CHe) reflects an increase in cardiovascular pulsation amplitude in V1 visual cortex during rotating checkerboard activation. Activation timing is marked with gray background, and stimulus video duration with light blue color. For **(A–D)**, *n* = 23. **(A)** IC1, representing primary visual cortex (V1), group-level mean signal increase of the original full-band signal (BOLD_FB_). **(B)** Bandpass filtered cardiac frequency signal, BOLD_Card_ in blue color signal reveals an oscillating cardiovascular pulsation and the envelope signal CHe in green color. **(C)** Normalized group mean time-series of BOLD_VLF_ response (± SE, black) shown with CHe (± SE, green) representing cardiovascular amplitude with a cross-correlation threshold of at least 0.3. The mean CHe time signal matches with the BOLD_VLF_ time domain signal and on average the CHe preceded the BOLD_VLF_ by 1.3 s. **(D)** An intra-voxel correlation map between CHe and BOLD_VLF_ voxel signals, with corresponding correlation coefficients color-coded in green.

The visual stimulus was displayed using a Nordic Neurolab^®^ MRI compatible InroomViewing Device (700 mm × 400 mm) positioned outside the scanner tube and viewed *via* the 32-channel head coil’s mirror (160 mm × 70 mm) that was positioned 15 cm above the subject’s eyes and driven by Neurobehavioral Systems Presentation software, which was triggered with the scanner T-pulse synchronizing the data acquisition with the stimulus. The visual stimulation video (125 s) consisted of a 5 s static cross at the beginning and four periods of visual stimulation (15 s each) showing a rotating checkerboard video in alternation with rest periods (15 s each) showing a static cross after each stimulus. The checkerboard rotations altered in direction and speed every second to minimize visual adaptation. Additionally, there was a 60 s rest period at the beginning of scanning and a 60 s rest period after the end of the stimulation video and continuing until the end of the scan, with presentation of a slightly smaller static cross. Non-stimulated time segments of 30 s from the beginning and 35 s from the end were excluded from all analyses (leaving the time segment depicted in Figure 1).

### Visual Activation

First, the preprocessed full 0–5 Hz band MREG data (henceforth designated as BOLD_FB_) was evaluated with respect to its portrayal of the visual activation and the overall signal change during visual stimulation. The first IC component (IC1), which covered the V1 primary visual cortex and contained the highest amount of signal variation, was used to show the activations. A group mean time-series of the IC1 area is shown in Figure 1A. Furthermore, we calculated the mean lag values for the onset (time after stimulus start until the first signal increase) and the end of activations (time after stimulus end until signal peak).

### Physiological Pulsation in Visual Cortex

One of the main goals of this study was to find whether patterns in physiological activity (respiratory and cardiac) exist in the visual cortex during visual task performance, and how such high frequency components may relate to the conventional BOLD response. First, the BOLD_FB_ datasets (0–5 Hz) were band-pass filtered to the very low (BOLD_VLF_, 0.01–0.1 Hz) frequency band. Then, based on the individual frequency peaks found in each subject, the datasets were also band-pass filtered to respiratory (BOLD_Resp_, mean 0.25 ± 0.07 Hz) and cardiac (BOLD_Card_, mean 1.01 ± 0.16 Hz) frequency bands. Individual frequencies were calculated from the BOLD_FB_ datasets, an approach which has been shown to be accurate compared to standard physiological measurement methods (Tuovinen et al., 2020). To prevent cardiorespiratory modulation, we used a bandwidth of 0.1 Hz (peak ± 0.05 Hz) for the physiological bands (Raitamaa et al., 2021). Then, the upper envelopes of the BOLD_Resp_ and BOLD_Card_ datasets were extracted from each voxel time-series. This was accomplished by finding the signal peaks and fitting a curve to the filtered data using cubic interpolation (MATLAB *findpeaks*, *spline, ppval*). Subsequently, we refer to these envelope signals as the respiratory pulsation envelope (Rpe) and cardiovascular hemodynamic envelope (CHe), respectively.

In order to quantify the correlation of the BOLD response time domain behavior with the physiological pulsation amplitude, we cross-correlated the CHe and Rpe voxel time-series against the same BOLD_VLF_ voxel time-series. These calculations were done using MATLAB *xcorr* with sequence normalization (parameter “*coeff*”), with a maximum allowed time lag of 5 s. We further examined the overall mean lag between CHe and BOLD_VLF_ signals from same voxels in the IC1 area, where the individual time-series with a correlation coefficient > 0.3 were selected from all subjects.

### Signal Source Analyses

We ran FSL FEAT for the BOLD_FB_ datasets to reveal the activation evident in visual cortex when using the full band data. In the first-level analysis, we configured a general linear model with a custom-designed square wave signal that matched the visual stimulus timing. Double-Gamma HRF convolution with temporal filtering was used to estimate the signal for a more realistic fit with the stimulus response. Then, a higher-level statistical analysis was run at the default setting.

In addition to BOLD_FB_, group-level ICA was also performed for BOLD_VLF_ and CHe datasets to see whether similar independent visual components could be found in them. We arbitrarily selected four distinct components within the V1 and higher order visual cortex from the BOLD_FB_ run, which were then correlated to all BOLD_VLF_ and CHe components using FSL *fslcc*. We then selected from the BOLD_VLF_ and CHe data the components with the highest spatial correlation to BOLD_FB_. These obtained spatial components were then refined using *fslmaths* with a threshold z-score value of 5. Finally, for visualization, the mean time-series of the remaining component areas were extracted using *fslmeants*.

### Amplitude and Lag Analyses

Using the previously obtained FEAT area as a mask, we calculated amplitude maps for BOLD_VLF_ and CHe to reveal which areas throughout the visual cortices had the highest amplitude during visual stimulation. This was accomplished by finding the peak time-points from each of the four stimulus periods and calculating their average for each voxel. To determine the similarity of the amplitude maps, spatial correlation values between the obtained BOLD_VLF_ and CHe amplitude maps were calculated using *fslcc*.

We also calculated the spatial lag distributions between BOLD_VLF_ and CHe across the FEAT mask area. Lag values were calculated using MATLAB *xcorr* with sequence normalization (parameter “*coeff*”) and a maximum allowed lag of 5 s. Based on the correlation range seen in Figure 1, we used a correlation coefficient threshold value of 0.3.

### Statistical Tests

**FIGURE 2:**
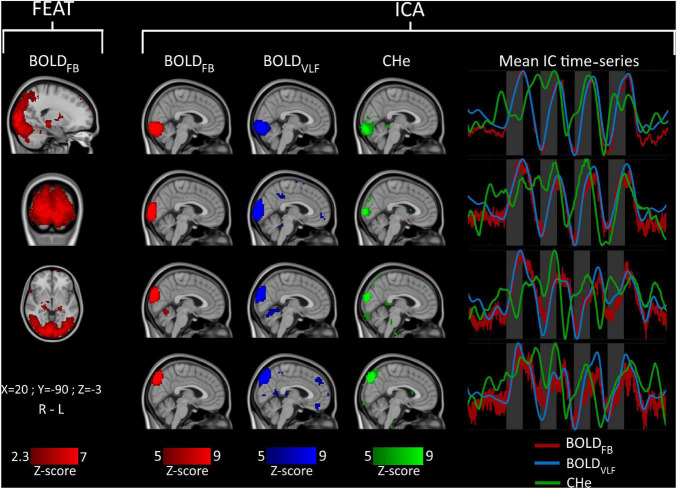
Group-level (*n* = 23) FEAT (left) and independent component (IC) signal source analysis (right). Mean IC time-series of component regions are shown on the right side of the figure in matching colors. Visual stimuli are marked with gray bars.

To see whether the visual cortex is the prevalent area of activation, a non-parametric one-sample group mean test (5,000 permutations) implemented in FSL *randomize* was used for the testing of previously obtained correlation maps between BOLD_VLF_ and CHe. The FEAT activation area (Figure 2) was used as mask.

## Results

### Cardiovascular Pulsatility Correlates With V1 Visual Cortex Blood Oxygen Level Dependent Activation

The full band BOLD signal (BOLD_FB_) was used to evaluate the overall group-level signal change in the central visual cortex (IC1, representing V1) prior to filtering (Figure 1A). The activations were observed across the four stimulation periods, with the first activation showing the highest signal change, and the later activations having declining amplitudes. The mean onset for the signal increase was 5.0 s after the beginning of a stimulation period, whereas the end of the signal increase (peak point) occurred at a mean of 3.7 s after the end of stimulation.

The extraction process of the CHe signal is shown in Figure 1B. Using the CHe as a proxy for brain activation, we were able to detect a signal that matched with the visual stimulus paradigm (Figure 1C). The correlation analysis showed that the highest within-voxel temporal correlations between BOLD_VLF_ and the CHe-contrasts were mostly concentrated within the IC1 area but extending slightly into its margins (Figure 1D). The correlation coefficient of CHe with BOLD_VLF_ was in the range 0.3–0.4. However, there was a high variance between subjects, ranging from negative correlation values up to + 0.8.

CHe had a strong tendency to precede (1.3 ± 2.2 s) the BOLD_VLF_ signal in the V1. Generally, the smallest lag difference between the contrasts occurred during the first stimulus peak, while the lag difference increased during the three subsequent stimuli, as the BOLD_VLF_ response extended over a broader time, i.e., the BOLD_VLF_ responses lasted longer on average than the CHe signal responses. The BOLD_VLF_ response widened as a function of time after the activation, but the CHe followed the activation onset and end timings more precisely in successive stimuli, c.f. Figure 1C.

### CHe Forms an Independent Brain Signal Source Similar to Blood Oxygen Level Dependent Signal Sources

As the response to visual stimulus was conspicuous in BOLD_VLF_, and to a lesser extent in CHe, we wanted to see how well the FEAT analysis could detect the visual activation areas prior to filtering. FSL FEAT one-group analysis revealed a wide activation pattern with *z*-score > 2.3 (*p* < 0.01) across the extended visual cortex, covering the V1-V4 areas (Figure 2).

Similar spatial components were found in each of the group ICA runs (Figure 2). Notably, the BOLD_FB_ and BOLD_VLF_ had components with very high spatial correlation values (>0.9). In the case of CHe, there were spatially similar components, but of lower volume than those for BOLD_FB_ and BOLD_VLF_, and with correspondingly lower spatial correlation values (>0.6). The BOLD_FB_ and BOLD_VLF_ mean IC time-series matched well with the stimulation period timings, while CHe varied the most. However, the CHe time-series tended to precede the BOLD signals also in this analysis.

### CHe Correlates With BOLD_VLF_ in Central Visual Areas

**FIGURE 3:**
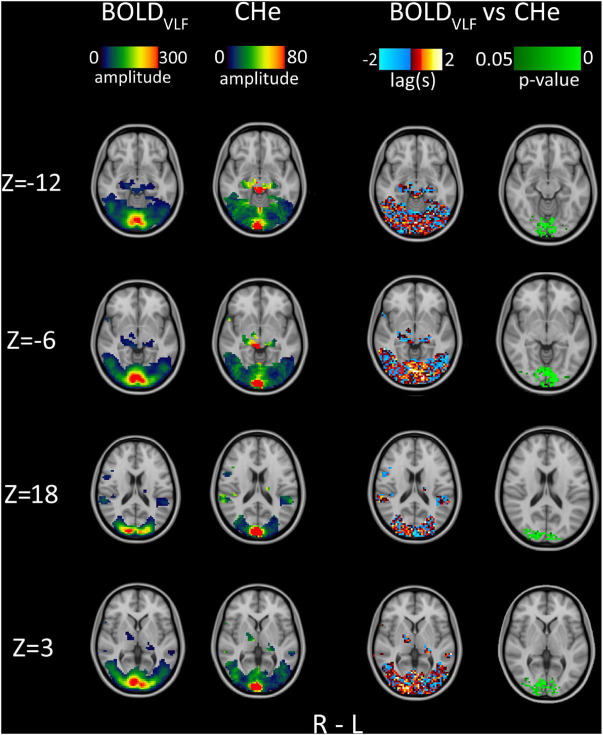
Group average (*n* = 23) amplitude, 1st column: BOLD_VLF_ amplitude map (relative percentage map), 2nd column: CHe amplitude map focusing near the posterior V1 area, in proximity to the draining sagittal sinus. 3rd column: Lag between BOLD_VLF_ and CHe voxel time-series. 4th column: Statistical significance (*p* < 0.05) of correlation values between BOLD_VLF_ and CHe voxel time-series within visual areas.

Areas with the largest amplitude (shown in the 1st and 2nd columns in Figure 3) matched partially between BOLD_VLF_ and CHe. The highest BOLD_VLF_ amplitude peaks were strictly confined to V1 and decreased toward the secondary visual areas. While there was a modest increase in the CHe amplitude in V1 areas, the largest amplitudes were concentrated near the sagittal sinus. The average spatial correlation between BOLD_VLF_ and CHe amplitude areas was 0.65 (± 0.06).

Since CHe seemed to offer the possibility to discern different neurovascular response (BOLD_VLF_) coupling times from hemodynamic inflow effects (CHe) of BOLD, we assessed the timing of CHe with respect to the brain activation BOLD_VLF_ signal. The warm yellow-red areas in the 3rd column in Figure 3 indicate areas where CHe preceded the BOLD_VLF_, and vice versa for the cold blue color. As can be discerned, the lag values of CHe were relatively stable only in the V1 area (Figure 3, *Z* = −6), where the BOLD_VLF_ also had the highest amplitude. In those regions, there was also a tendency for the CHe signal to precede the BOLD_VLF_ signal onset., c.f. Figure 3. Statistically, the significant (*p* < 0.05) CHe was located quite strictly within the V1 and V2 areas, thus overlapping slightly with the areas where CHe preceded the BOLD_VLF_ (Figure 3, 4th column).

### Respiratory Pulsation Envelope Depicts Respiratory Brainstem Centers but Not Visual Activity

**FIGURE 4:**
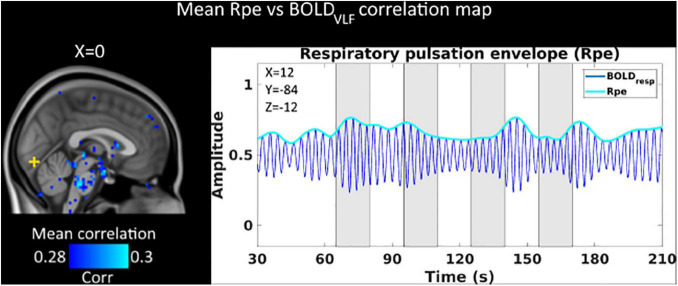
Mean (*n* = 23) within-voxel correlation map between BOLD_VLF_ and respiratory pulsation envelope (Rpe). We see within-voxel correlations between Rpe and BOLD_VLF_ in detecting respiratory modulations of the BOLD signal near the brainstem respiratory centers. There was no correlation between Rpe and BOLD_VLF_ signal in the activated visual cortex (yellow cross). Visual stimuli are marked with gray.

We also analyzed whether the respiratory pulsation envelope had any temporal relation to the visual stimulus. The voxel-by-voxel respiratory pulsation envelope (Rpe), either alone or in correlation analysis with the BOLD_VLF_ signal, did not reveal any significant association with the visual stimulus. On the other hand, the Rpe did correlate with BOLD_VLF_ near the pneumotaxic and apneustic respiratory centers of the brain stem, suggesting sensitivity to mapping of respiration related activity (Figure 4).

## Discussion

Our study shows that sensory activation of visual cortex increased cardiovascular brain pulsations in central V1 areas where the BOLD_VLF_ amplitude is also the largest. The cardiovascular hemodynamic envelope (CHe) showed that cardiovascular pulsatility increased immediately after the onset of the visual stimulus. The pulsatility increase preceded the canonical BOLD response, while respiratory related brain pulsations did not track the visually cued activity.

We show for the first time an increasing cardiovascular pulsatility mapping simultaneously with canonical BOLD responses in the human visual cortex. Mosso (1880) first described an increase in human brain pulsatility during an activation task, followed later by Roy and Sherrington (1890) and Berger (1901) in experimental animals. Those early studies were done invasively through an open skull injury or by trepanation. More than a century later, fast fMRI methods enable non-invasive detection of analogous phenomena in human brain (Lin et al., 2012; Assländer et al., 2013; Boyacioğlu and Barth, 2013; Posse et al., 2013; Agrawal et al., 2020) and also in rodents (Yu et al., 2014).

During neuronal activation, the regional vasodilation driven by neuro-astrocytic signaling leads to increased hemodynamic inflow of oxygenated blood to venules (a.k.a activation hyperremia) depending on activated area and species (Biswal et al., 2003; Kucewicz et al., 2007; MacVicar and Newman, 2015; Drew et al., 2020; Grubb et al., 2020). Neurovascular unit signals the precapillary sphincter to relax and as the tone of the smooth muscle cells around arterioles weakens momentarily, the perfused capillary bed of the dilated arteriole receives more pulsatile flow (Figure 1B). In our data the envelope of the cardiovascular hemodynamic signal, the CHe, represents a marker of the pulsatility that follows the relaxation of the precapillary sphincters. CHe can be seen as an early surrogate for neuronal activity since it preceded the BOLD_VLF_ response on average by some 1.3 (± 2.2) s, c.f. in Figure 1C. Previously, human transcranial ultrasound studies of rapid sampling rate were able to capture increased brain pulsatility in the visual cortex during visual brain stimulation (Kucewicz et al., 2007). A mouse whisker stimulus study with multiphoton microscopy showed that the regional blood flow pulsatility also increased within the dilating precapillary sphincter and within blood vessels downstream from the sphincter (Grubb et al., 2020). Thus, the present findings with fast fMRI are not entirely without precedent in other physiological settings.

In mice cortices, the activated cortical area, neuronal activity induces dilation of precapillary smooth muscle sphincters, allowing the enhanced pulsations to deliver more blood to the tissue (Grubb et al., 2020). In addition, the pulsatile nature of blood flow has itself been shown to increase NO-mediated vasodilation, which further facilitates the downstream vasodilatory brain responses in the vascular trees (Nakano et al., 2000). The increased vasodilation in turn causes increased pulsatility of the relaxed vessel walls of arteries and arterioles in concert with declining smooth muscle tonus. An increased vessel wall pulsation has been shown to increase further the penetrance of the interior pulsatile effect from the intraluminal space into the perivascular space, thus augmenting the CSF flow through the perivascular structures and into the parenchyma (Xie et al., 2013; Mestre et al., 2018).

The fMRI BOLD signal response knowledge indicates that in activated brain regions, an increase in fMRI T2*weighted signal intensity that is sensitive to the reducing deoxyhemoglobin concentrations and increasing blood volume in the draining venules after some 3–5 s (Ogawa and Lee, 1990; Bandettini et al., 1992; Ogawa et al., 1992; Buxton, 2012). In agreement to the previous data, the detected BOLD_FB_ signal response peak lag in the V1 was on average 3.7 s as measured from the end of activation (Figure 1A). Downstream from the activated pulsatile capillary segments, the venules become dilated by increased cerebral blood flow and volume and the paramagnetic deoxyhemoglobin becomes increasingly replaced by diamagnetic oxygenated blood, resulting less dephasing effects and stronger T2* weighted. In this study, the inter-stimulus interval was brief to minimize the scan length, and thus the carryover of post-stimulus undershoots from previous stimuli overlap and increase following the negative dip of the BOLD response, which renders the lag inferences more reliable from the BOLD_VLF_ signal peak rather than from the first emerging initial signal change of 5 s (Figure 1C).

The remaining time differences suggest that the time lag from stimulus onset in CHe was about 2.4 s (i.e., BOLD peak 3.7 vs. CHe 1.3 s overall precedence) from the start of neuronal activation to the detected increase in cardiovascular CHe pulsation. Similar BOLD based techniques showed that an inverted (flow-weighted) carotid artery BOLD signal precedes the global BOLD signal by an average of 2.7 s, which is also comparable to our results, given the anatomical distance of the carotid from the visual cortex (Amemiya et al., 2020). In a pioneering study, Biswal and Pathak used combined BOLD and dynamic contrast enhanced (DCE) MRI measurement and detected with a correlation analysis a neurovascular (neuronal + vasomotor) response delay of 0.7 (± 0.6) s and an inflow (transit) delay of 2.3 (± 1.1) s in the human motor cortex (Biswal et al., 2003).

In neurophysiological terms, the increased cardiovascular pulsation in activated brain tissue promotes increased convection of brain tissue metabolites in the paravascular Virchow-Robin space (Rennels et al., 1985; Mestre et al., 2018; Yokoyama et al., 2021). In the present study, we obtained evidence in brain of healthy awake volunteers that cardiovascular pulsatility does indeed increase somewhat before the BOLD response as occurs after activation-induced vasodilation. Two recent studies indicated that a harmonic 0.1 Hz vasomotion induced by visual activation itself also increases interstitial metabolite clearance (Kedarasetti et al., 2020; van Veluw et al., 2020). Importantly, during visual activation, the repeated vasodilatations occur *locally* in the visual cortex blood vessel walls, which likely enables regionally more effective coupling of the pulsation to perivascular metabolite convection.

We argue that the increased cardiovascular pulsation amplitude in the visual cortex during stimulation could depict an increase in the force driving the (para)vascular convection of metabolites, which may serve the increasing metabolic demands during brain activation, as supported by localized vasodilatory effects (Mestre et al., 2018; Grubb et al., 2020). Furthermore, in line with van Veluw et al. (2020), the activation-induced changes in vasomotion and subsequently in regional cerebral blood volume may also transform tissue hydrodynamics so as to increase metabolite transfer (Kedarasetti et al., 2020). The activation-induced ballooning of the veins, which manifests later as the BOLD response, marks redistribution in the blood compartments and might itself facilitate metabolite transfer gradients. In practice, the vasomotor-driven metabolite transfer requires a porous connection between the perivascular space and interstitium, which could be mediated by AQP4 water channels in astrocytic endfeet (Nedergaard, 2013). As these hydrodynamic changes are closely coincident during activation, the vessel wall pulsatility and other forms of vasomotion may well work synergistically, rather than exclusively serving brain tissue homeostasis.

### CHe as a Physiological Imaging Contrast

During the past few decades, functional neuroimaging research has focused on detecting shifts in the baseline blood flow, metabolism in positron emission tomography (PET) or oxygenation as governed by CBF/V or OEF/CMRO_2_ changes. Moreover, the physiological pulsatility has been considered as a nuisance obscuring the indirect hemodynamic BOLD responses to neuronal activity, and several tools have been developed in order to remove these pulsatile changes (Dagli et al., 1999; Salimi-Khorshidi et al., 2014; Tong and Frederick, 2014; Agrawal et al., 2020). This emphasis was also partially due to the lack of spatiotemporal accuracy of neuroimaging methods, which have only recently attained levels sufficient to image individual cardiovascular impulses with high precision (Lin et al., 2012; Posse et al., 2013; Kiviniemi et al., 2016).

However, instead of being a nuisance signal to be removed, the cardiovascular brain pulsatility can offer new information of the neurovascular function in the brain cortex. The fast CHe response offers a new information channel for estimating neurovascular lag structures, as the CHe response precedes the BOLD_VLF_ response in the V1 area. In the V1 central visual area, the largest BOLD_VLF_ response partially coincided with the time of maximal CHe amplitude (Figure 3). Outside the most highly activated V1 region, the CHe analysis produced a relatively random pattern of lags with high inter-subject variability, when compared to stimulus timing and to individual voxel BOLD signals (Figure 3). In addition, the correlation of CHe signal to the BOLD_VLF_ response in the central visual cortex was on average only around 0.3 (Figure 1D). However, the CHe correlated significantly with BOLD_VLF_ in the central V1 areas (Figure 3) while the correlation between Rpe and BOLD_VLF_ showed no link to the visual cortex (Figure 4). The CHe seems to have marked variability, such that elevations in the pulsation are seldom discernible prior to the onset of the stimulus. Thus, the CHe is clearly not as specific to increased neuronal activity as is the BOLD response but is seemingly influenced by yet unknown other sources of signal variance. These unknown sources may be influential factors while pursuing un-identified forms of neurovascular interactions such as pathology.

Tools like ICA (Calhoun et al., 2001; Kiviniemi et al., 2003; Beckmann et al., 2005) are able to depict functional networks based on independent pulsatility features more confined than BOLD signal (Figure 2). The CHe contrast is an independent signal source, which reflects the similar functional network distributions, as shown in a previous study of physiological signal rather than VLF BOLD (Akin et al., 2017), c.f. Figure 2. Therefore, CHe seems to be an independent brain phenomenon embedded within resting state networks, and not an epiphenomenon or trivial consequence of activation hyperemia driven by VLF. Furthermore, CHe did in fact precede the BOLD_VLF_ signal increase in the most highly activated visual areas. Further research is required to understand better the drivers of cardiovascular brain CHe pulsatility, which may subserve multiple purposes in relation to maintenance of physiological homeostasis during activation (Mestre et al., 2018).

### Limitations

The detected CHe contrast is formed from the envelope of the peaks of cardiovascular brain pulsations. It follows that the individual heart rate dictates the inherent, physiological “sampling rate” of the time domain signal. Thus, CHe can be only accurate within some range of heart rate and is bound to have some variability stemming from arterial spatial distribution changes, local phase shifts in the pulsation, as well as from the known heart rate variability. The slower BOLD_VLF_ signal has a larger amplitude, smaller variance, and less sensitivity to beat-to-beat heart rate variations, c.f. Figure 3. As such, the classical BOLD signal clearly outperforms the CHe contrast with respect to sensitivity and for delineating the activated visual areas, Figures 2, 3.

The CHe signal mainly mapped to near the V1 area, where the BOLD_VLF_ signal increase had the highest amplitude. This suggests that the BOLD amplitude could depend on the amplitude of vascular pulsation controlled by regional vasomotor dilations/contractions. However, our analyses indicated rather low correlations between CHe and the BOLD_VLF_ signal. Spatial resolution, cortical contours, and/or regional pulsatility phase fluctuations could all be factors interfering in the detection of the expected correlation structure by present methods.

The prolonged long visual stimulation of 15 s caused the time signals to increase in amplitude during the entire stimulation period; this phenomenon interfered in the analysis of peak timing, which usually entails short 1–2 s stimuli. Furthermore, a relatively brief inter-stimulus interval seems to alter the BOLD time signal as a function of stimulus. Shorter bursts of stimulation and varied inter-stimulation intervals might thus have given robuster approximations for the BOLD_VLF_ lag between stimulations. However, a shorter duration of activation may be less fit for evaluating the cardiorespiratory pulsation responses to visual stimuli. The alternating rotational directions of the checkerboard stimulus may also add variance to the BOLD response, as this may have induced negating responses in opposite visual fields during reversal of the rotation direction. Additionally, we chose non-stimulated rest segments also lasting 15 s between the visual stimuli. This might not have allowed sufficient time for the post-stimulation signal to drop to baseline, which could therefore have negatively biased the responses to subsequent stimuli. Nevertheless, our use of longer stimulation periods enabled the detection of a brain-wide activation pattern and may also have been beneficial for correlating the BOLD_VLF_ and CHe signals.

## Conclusion

We found evidence supporting a model in which the regional brain hyperemic activation response coincides with increased cardiac but not respiratory brain pulsations elsewhere implicated as drivers of CSF flow. During a visual activation task, physiological cardiovascular brain pulsation signals increased in the V1 and V2 cortical brain areas, where the BOLD_VLF_ responses also had the largest amplitude. Furthermore, the CHe signal activation seemed to precede the BOLD_VLF_ response by more than 1 s, and showed a similar, clear response to stimulation as seen in BOLD_VLF_. However, further research is required to obtain a comprehensive understanding of the drivers of the CHe pulsation signal and its sources of variance.

## Data Availability Statement

The original contributions presented in the study are included in the article/supplementary material, further inquiries can be directed to the corresponding author/s.

## Ethics Statement

The studies involving human participants were reviewed and approved by Regional Ethics Committee of Northern Ostrobothnia Hospital District in Oulu University Hospital. The patients/participants provided their written informed consent to participate in this study.

## Author Contributions

VKi, VKo, and NH designed the study. HH, NH, and VKo collected the data. NH and LR analyzed the data. VKi, NH, JT, VKo, JK, MJ, HH, TT, and VR wrote the manuscript. All authors contributed to the article and approved the submitted version.

## Conflict of Interest

The authors declare that the research was conducted in the absence of any commercial or financial relationships that could be construed as a potential conflict of interest.

## Publisher’s Note

All claims expressed in this article are solely those of the authors and do not necessarily represent those of their affiliated organizations, or those of the publisher, the editors and the reviewers. Any product that may be evaluated in this article, or claim that may be made by its manufacturer, is not guaranteed or endorsed by the publisher.
